# Supplementary material for: HIVprotI: an integrated web based platform for prediction and design of HIV proteins inhibitors
Source: J Cheminform. 2018 Mar 9;10:12. doi: 10.1186/s13321-018-0266-y (PMC5845081; doi:10.1186/s13321-018-0266-y)
Supplement: Supplementary file 2 — Additional file 2. Source code of HIVProtI web server. [file 13321_2018_266_MOESM2_ESM.zip › HIVprotI_Source-code/hivprotI/weka-3-6-6/doc/weka/classifiers/functions/IsotonicRegression.html]

IsotonicRegression


---


|  |  |  |
| --- | --- | --- |
| **Overview** \| **Package** \| **Class** \| **Tree** \| **Deprecated** \| **Index** \| **Help** \| **Weka's home** | |  |
| **PREV CLASS**   **NEXT CLASS** | **FRAMES**    **NO FRAMES**     **All Classes** |  |
| SUMMARY: NESTED \| FIELD \| CONSTR \| METHOD | DETAIL: FIELD \| CONSTR \| METHOD |  |


---


## weka.classifiers.functions Class IsotonicRegression

```
java.lang.Object
  weka.classifiers.Classifier
      weka.classifiers.functions.IsotonicRegression
```

**All Implemented Interfaces:**: java.io.Serializable, java.lang.Cloneable, CapabilitiesHandler, OptionHandler, RevisionHandler, WeightedInstancesHandler

---

``` public class IsotonicRegression extends Classifier implements WeightedInstancesHandler ```

Learns an isotonic regression model. Picks the attribute that results in the lowest squared error. Missing values are not allowed. Can only deal with numeric attributes.Considers the monotonically increasing case as well as the monotonicallydecreasing case


Valid options are:

```
 -D
  If set, classifier is run in debug mode and
  may output additional info to the console
```

**Version:**
:   $Revision: 5523 $

**Author:**
:   Eibe Frank (eibe@cs.waikato.ac.nz)

**See Also:**: Serialized Form

---

**Constructor Summary**

`IsotonicRegression()`


| **Method Summary** | |
| --- | --- |
| `void` | `buildClassifier(Instances insts)`             Builds an isotonic regression model given the supplied training data. |
| `double` | `classifyInstance(Instance inst)`             Generate a prediction for the supplied instance. |
| `Capabilities` | `getCapabilities()`             Returns default capabilities of the classifier. |
| `java.lang.String` | `getRevision()`             Returns the revision string. |
| `java.lang.String` | `globalInfo()`             Returns a string describing this classifier |
| `static void` | `main(java.lang.String[] argv)`             Main method for testing this class |
| `java.lang.String` | `toString()`             Returns a description of this classifier as a string |

**Methods inherited from class weka.classifiers.Classifier**

`debugTipText, distributionForInstance, forName, getDebug, getOptions, listOptions, makeCopies, makeCopy, setDebug, setOptions`

**Methods inherited from class java.lang.Object**

`equals, getClass, hashCode, notify, notifyAll, wait, wait, wait`

**Constructor Detail**

### IsotonicRegression

```
public IsotonicRegression()
```


**Method Detail**

### globalInfo

```
public java.lang.String globalInfo()
```

:   Returns a string describing this classifier

    :   **Returns:**: a description of the classifier suitable for displaying in the explorer/experimenter gui

---


### classifyInstance

```
public double classifyInstance(Instance inst)
                        throws java.lang.Exception
```

:   Generate a prediction for the supplied instance.

    :   **Overrides:**: `classifyInstance` in class `Classifier`
    :   **Parameters:**: `inst` - the instance to predict. **Returns:**: the prediction **Throws:**: `java.lang.Exception` - if an error occurs

---


### getCapabilities

```
public Capabilities getCapabilities()
```

:   Returns default capabilities of the classifier.

    :   **Specified by:**: `getCapabilities` in interface `CapabilitiesHandler` **Overrides:**: `getCapabilities` in class `Classifier`
    :   **Returns:**: the capabilities of this classifier **See Also:**: `Capabilities`

---


### buildClassifier

```
public void buildClassifier(Instances insts)
                     throws java.lang.Exception
```

:   Builds an isotonic regression model given the supplied training data.

    :   **Specified by:**: `buildClassifier` in class `Classifier`
    :   **Parameters:**: `insts` - the training data. **Throws:**: `java.lang.Exception` - if an error occurs

---


### toString

```
public java.lang.String toString()
```

:   Returns a description of this classifier as a string

    :   **Overrides:**: `toString` in class `java.lang.Object`
    :   **Returns:**: a description of the classifier.

---


### getRevision

```
public java.lang.String getRevision()
```

:   Returns the revision string.

    :   **Specified by:**: `getRevision` in interface `RevisionHandler` **Overrides:**: `getRevision` in class `Classifier`
    :   **Returns:**: the revision

---


### main

```
public static void main(java.lang.String[] argv)
```

:   Main method for testing this class

    :   **Parameters:**: `argv` - options


---


|  |  |  |
| --- | --- | --- |
| **Overview** \| **Package** \| **Class** \| **Tree** \| **Deprecated** \| **Index** \| **Help** \| **Weka's home** | |  |
| **PREV CLASS**   **NEXT CLASS** | **FRAMES**    **NO FRAMES**     **All Classes** |  |
| SUMMARY: NESTED \| FIELD \| CONSTR \| METHOD | DETAIL: FIELD \| CONSTR \| METHOD |  |


---
